# Creating implementable clinical practice guidelines: the 2020 Focused Updates to the National Heart, Lung, and Blood Institute’s Asthma Management Guidelines

**DOI:** 10.1186/s43058-023-00417-3

**Published:** 2023-03-31

**Authors:** Susan T. Shero, Neyal J. Ammary-Risch, Edwin A. Lomotan, Russell E. Mardon, Maria Michaels

**Affiliations:** 1 National Heart Lung and Blood Institute, Bethesda, MD USA; 2 Agency for Healthcare Research and Quality, Rockville, MD USA; 3 Westat, Rockville, MD USA; 4 Centers for Disease Control and Prevention, Atlanta, GA USA

**Keywords:** Implementability, Guidelines, Clinical decision support, Asthma

## Abstract

**Background:**

The 2020 Focused Updates to the Asthma Management Guidelines: A Report from the National Asthma Education and Prevention Program Coordinating Committee Expert Panel Working Group provides the first new clinical practice recommendations from the National Heart, Lung, and Blood Institute (NHLBI) since the previous 2007 asthma management guidelines. Guideline implementability was a high priority for the expert panel, and many approaches were undertaken to enhance the implementability of this clinical guideline update.

Within the report, specific implementation guidance sections provide expanded summaries for each recommendation to quickly assist users. The implementation guidance incorporates findings from NHLBI-sponsored focus groups conducted with people who have asthma, caregivers, and health care providers. The findings were used to identify the types of information and tools that individuals with asthma, their caregivers, and their health care providers would find most helpful; ensure that the new asthma guidelines reflect the voices of individuals with asthma and their caregivers; and identify potential barriers to uptake by individuals with asthma and their caregivers. The expert panel used a GRADE-based approach to develop evidence-to-decision tables that provided a framework for assessing the evidence and consideration of a range of contextual factors that influenced the recommendations such as desirable and undesirable effects, certainty of evidence, values, balance of effects, acceptability, feasibility, and equity. To facilitate uptake in clinical care workflow, selected recommendations were converted into structured, computer-based clinical decision support artifacts, and the new recommendations were integrated into existing treatment tables used in the 2007 asthma management guidelines, with which many users are familiar. A comprehensive approach to improve guidelines dissemination and implementation included scientific publications, patient materials, media activities, stakeholder engagement, and professional education.

**Conclusion:**

We developed evidence-based clinical practice guideline updates for asthma management focused on six topic areas. The guideline development processes and implementation and dissemination activities undertaken sought to enhance implementability by focusing on intrinsic factors as described by Kastner, Gagliardi, and others to produce usable, adoptable, and adaptable guidelines. Enhanced collaboration during guideline development between authors, informaticists, and implementation scientists may facilitate the development of tools that support the application of recommendations to further improve implementability.

Contributions to the literature

- Use of the GRADE framework for the guideline updates facilitated implementability by considering factors such as feasibility but required technical support by a methodology team.
- Input from patients, caregivers, and providers is an important factor in understanding information needs that can guide the development of implementable recommendations. A need for greater specificity in the recommendations was noted post-release through discussions and questions at scientific conferences and during use case development for CDS. Guideline development efforts could benefit by incorporating informatics and implementation perspectives in guideline development panels as proposed in the future state of Adapting Clinical Guidelines for the Digital Age.

## Background

Clinical practice guidelines (CPGs) are statements that include recommendations intended to optimize patient care, informed by a systematic review of evidence and an assessment of the benefits and harms of alternative care options [1]. Although many CPGs exist and more continue to be developed, their uptake and implementation in clinical settings are less than ideal, likely related to a variety of factors [1, 2] which may include those related to the practice setting such as characteristics of the implementation strategies, professionals, patients, and environment (extrinsic), and those connected to the CPG itself (intrinsic) [3]. This paper focuses primarily on intrinsic factors. Implementability has been described as the characteristics or features of the guideline and/or recommendations that promote or enhance their use [4]. Kastner and colleagues [3] reported that guideline implementability is associated with both how the guideline content is created and the effective communication of that content. Gagliardi et al. [5] developed a guideline implementability framework that consists of 22 elements organized around the domains of adaptability, usability, validity, applicability, communicability, accommodation, implementation, and evaluation. Here we discuss updated approaches taken to enhance implementability of the 2020 Focused Updates to the Asthma Management Guidelines: A Report from the National Asthma Education and Prevention Program Coordinating Committee Expert Panel Working Group [6] which provided the first new asthma clinical practice recommendations from the National Heart, Lung, and Blood Institute (NHLBI) since the previous 2007 asthma management guidelines (Expert Panel Report [EPR]-3) [7]. Many of these approaches address the elements associated with the domains in Gagliardi et al.’s guideline implementability framework and incorporate elements of the Adapting Clinical Guidelines for the Digital Age initiative described below.

### Use of the GRADE framework to enhance implementability

The Grading of Recommendations Assessment, Development, and Evaluation (GRADE) methodology is an internationally accepted framework for determining the quality or certainty of evidence and the direction and strength of recommendations based on this evidence [8]. The Expert Panel that developed the 2020 Focused Updates used the GRADE approach to review the evidence, create evidence profiles for critical and important outcomes, develop evidence-to-decision tables, and write recommendation statements [6]. The GRADE approach balances the likelihood of potential effects stemming from a recommendation, both desirable and undesirable, with the certainty of the evidence supporting these likelihoods. Importantly, an integral part of the evidence-to-decision framework is an assessment of feasibility—is the recommendation feasible to implement? If there are common barriers to implementation that would be difficult to overcome, then that recommendation is less clinically relevant for the target audience of the CPG, and the less likely it is that it should be recommended [9]. The assessment of feasibility depends upon the target audience for the CPG as it considers the resources available in typical clinical settings for that audience. The 2020 Focused Updates explicitly considered implementability in the development of the recommendations by building upon the GRADE methodology and incorporating clinically relevant implementation considerations into the guideline.

### Implementation guidance within the guideline and treatment diagrams

An important feature of the presentation of each recommendation in the 2020 Focused Updates is the Implementation Guidance. The Implementation Guidance sections are written for practicing clinicians and incorporate the findings from NHLBI-sponsored focus groups with people with asthma, caregivers, and providers. They appear immediately following each recommendation statement and include a concise explanation of the asthma recommendation (Clinician’s Summary) that clearly states the patient population, diagnostic history, and other relevant details of the clinical situations to which the recommendation applies, along with a summary of the rationale and evidence for the recommendation. These sections also include the following information:

- Explanations of the population most likely to benefit from the asthma recommendation, as well as any populations to which the recommendation does not apply.
- Issues that clinicians should discuss with their patients as part of the shared decision-making process as it relates to asthma care. These may relate to tradeoffs between risks and benefits that depend upon patient preferences and individual situations.
- Other topic-specific considerations such as limitations in the evidence supporting the recommendations, feasibility considerations, and other cautions or considerations in implementing the asthma recommendation.

While the 2020 Focused Updates address a select set of priority topic areas^1^ and research questions, the Expert Panel recognized the need to integrate the new evidence-based recommendations into the existing comprehensive approach to asthma care familiar to clinicians from previous guidelines (Expert Panel Report-3 [7]) to enhance implementability. The main strategy for doing this was to update the EPR-3 step treatment diagrams that illustrate the stepwise approach for asthma management. These diagrams, found in both EPR-3 and the Focused Updates, are meant to assist and not replace the clinical decision-making required for individual patient management and the input from individuals with asthma about their preferences.

In updating the step diagrams, the Expert Panel used some of the definitions and assumptions from EPR-3 and retained the EPR-3 recommendations that were not addressed in the 2020 Focused Updates. The structure of the step diagrams is intended to support implementability by reducing the cognitive complexity for the clinician of integrating information from the patient and the EHR when applying the recommended asthma management steps. There are three updated step diagrams: one for ages 0–4 years, one for ages 5–11 years, and one for ages 12 and older. Within each step, there are preferred options for the best management choices supported by the evidence along with, in many cases, alternative options. These alternatives are management strategies that may be less effective or have more limited evidence than the preferred options. Clinicians and patients may choose the alternative treatments if individuals with asthma are currently receiving this therapy and their asthma is under control, if the preferred treatments are not available or are too costly, or if an individual with asthma prefers an alternative treatment. In the stepwise approach to therapy for asthma, the clinician escalates treatment as needed (by moving to a higher step) or, if possible, deescalates treatment (by moving to a lower step) once the individual’s asthma is well controlled for at least 3 consecutive months. Each step diagram contains several important footnotes that indicate which elements of the diagram represent new recommendations, as well as other important details about the use of the diagram.

### Leveraging the Adapting Clinical Guidelines for the Digital Age initiative

**Fig. 1:**
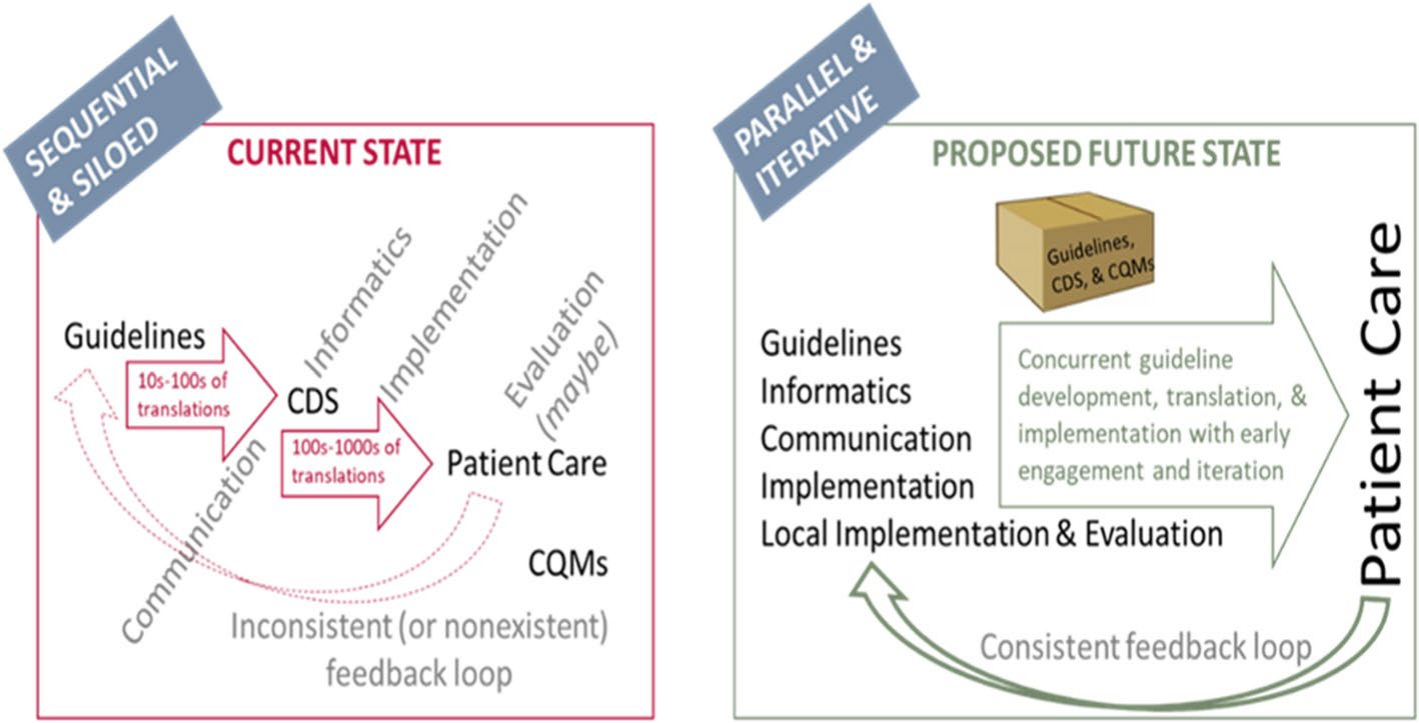
Redesigning guideline development and implementation: the integrated process [11]

To implement CPGs into patient care more easily, quickly, accurately, and consistently, the Centers for Disease Control and Prevention (CDC) has led a multi-partner initiative since 2018 on “Adapting Clinical Guidelines for the Digital Age” [10]. As part of this initiative, technical standards for developing and representing CPGs in computable form using Health Level 7’s (HL7’s) Fast Healthcare Interoperability Resources (FHIR®) standard were developed, balloted, and published (i.e., FHIR® Clinical Guidelines Implementation Guide [11], more commonly known as “CPG-on-FHIR®”). Likewise, an integrated process was created that redesigned guideline development and implementation so that downstream perspectives such as informatics, implementation, communication, and evaluation are part of guideline development from the outset (see Fig. 1). The combination of these technical standards and redesigned process results in a systematic, standards-based approach for co-developing written and computable guidelines [12] with implementation success across many clinical organizations as the top goal (e.g., creating shareable implementable CDS). Portions of these technical standards and redesigned process were leveraged for the 2020 Focused Updates when informaticists worked collaboratively with guideline developers to create computable artifacts for the asthma medication recommendations.

In the current state, the guideline development and implementation process is often sequential and siloed, where the development of the written guidelines, clinical decision support (CDS), and clinical quality measures (CQMs) are done separately from each other (Fig. 1). As depicted on the left side of Fig. 1, this approach leads to numerous translations of written guidelines into CDS artifacts and even more translations from CDS into patient care workflows, increasing the chance of not applying the CPG recommendations as intended. The development and implementation of CQMs are generally treated separately from patient care, making their use in evaluation of guideline uptake and outcomes limited. Evaluation and feedback loops, especially back to the guideline developers, are inconsistent or non-existent. Furthermore, producing guidelines only in written format may result in no specific implementation support within the clinical workflow, which would require human capacity to retain and correctly apply the knowledge contained within the recommendations. Increases in the scientific knowledge base, however, have exceeded human capacity [13]. Thus, synthesizing relevant person-specific data for the care of a patient in the context of one or more guidelines is cumbersome and likely to be incomplete without some form of cognitive support.

In the proposed future state, all perspectives are represented from the outset, working iteratively together to produce a “package” of products that includes the written and computable guidelines, CDS, CQMs, and potentially other derivatives of the computable guidelines that help implement the CPGs in patient care using a more integrated process. Feedback loops are more consistent and iterative, providing opportunity for better and more real-time monitoring of the CPGs in practice. As the science shows that updates or new CPGs are needed, the updates can be made modularly to the written and computable guidelines and the derivatives that support the implementation of the CPGs—an approach commonly referred to as “living guidelines” [14, 15]. Co-developing written and computable guidelines does not completely remove the potential for mistranslations into patient care. Computable guidelines and their derivatives still require implementation into clinical workflows at each healthcare organization. However, co-development does at least reduce the number of translations that create an increase in the probability that the recommendations are not implemented as intended. Further, by using an interdisciplinary approach from the beginning, implementation challenges that have historically been identified downstream (e.g., vaguely written recommendations that make it difficult to identify the best triggers for CDS, implementing alerts even if it is not the most effective way to apply the recommendations) [16, 17] could be discovered and addressed earlier in the process. This helps increase the likelihood of successful implementation into patient care.

### Clinical decision support to enhance dissemination and implementation

CDS can help make CPGs more useful for clinicians by incorporating them into electronic health records (EHRs) and other point-of-care decision support tools in a way that is shareable, standards-based, publicly available, and patient-centered. The translation of CPGs into computer-based CDS (e.g., derived from computable guidelines) has the potential to speed the uptake of evidence-based guidance into practice by putting the guidance at the fingertips of clinical teams at the point of care. However, the process of converting guideline recommendations into CDS remains a laborious and inefficient one because it often must be customized to meet the needs of individual healthcare systems [18]. The cost of redundant effort in translating guideline-based care into CDS across the US healthcare system has been estimated at $25 billion [18]. The Agency for Healthcare Research and Quality (AHRQ) has invested in a program dedicated to advancing public, web-based infrastructure to make shareable, interoperable CDS more accessible and widespread [19]. The infrastructure, called CDS Connect, includes an online repository of publicly available CDS resources or “artifacts” as well as tools for authoring standards-based CDS logic for implementation in EHRs and other technologies. NHLBI is among the Federal partners that have contributed to the CDS Connect repository by funding the development of a series of CDS artifacts related to the medication recommendations in the 2020 Focused Updates. At the time of the publication of these new CDS artifacts in CDS Connect in mid-2021, there were no asthma management tools in the CDS Connect library [20], a crucial gap. The development of these new CDS tools increases the potential for dissemination and implementation of evidence-based asthma CPGs through systematic, standards-based (i.e., using CPG-on-FHIR®), and replicable CDS development and deployment.

### Insights from people with asthma, caregivers, and healthcare providers

Understanding how people with asthma, their caregivers, and healthcare providers make treatment decisions, use CPGs, and prefer to be communicated with is critical for delivering high-quality care. Prior to the completion and launch of the 2020 Focused Updates, NHLBI’s *Learn More Breathe Better®* program conducted formative research to help inform the development of new educational materials, which can also be used to facilitate CDS implementation. This work included 10 focus groups with adults with asthma, caregivers of children ages 12 and younger with asthma, and 11 in-depth interviews with healthcare providers who treat asthma to better understand their communication needs. All were conducted using virtual data collection methods (telephone and online platforms). Focus group participants were English- and Spanish-speaking, lower- to lower-middle-income (< $50 k annual household income) adults with asthma (*n* = 27), and adult caregivers of children with asthma (*n* = 26). Few differences were found in the perspectives of adults with asthma versus caregivers and English versus Spanish speakers.

This qualitative research provided insight into the outcomes that patients and caregivers described as most important to them; factors that affect treatment choices; preferences for medication type and scheduling; and their views on allergy reduction, immunotherapy, and bronchial thermoplasty. The most desired outcome for patients and caregivers is relief from asthma symptoms. Factors that affect their treatment decision-making include cost and insurance coverage, safety, side effects, benefits, success rates, and asthma severity. Inhaled medications are preferred over pills or liquids because they are perceived to be easier to take or administer and be faster acting. Taking action to reduce allergens in the home was a common practice cited, with a majority reporting the use of mattress and pillow covers, vacuuming and dusting regularly, removing curtains and mold, and controlling for pests. Very few participants said they would stop their current allergen reduction efforts even if those efforts are proven to be ineffective. Generally, awareness of immunotherapy was low to moderate and Spanish-speaking adult patients were more receptive to bronchial thermoplasty. All participants wanted more information about treatment risks, side effects, and success rates. In parallel to the release of the updated Guidelines, *Learn More Breathe Better* created fact sheets to address each of the updated focus areas to help patients better understand different treatment options. These are freely available as part of a digital toolkit that supports promotion and implementation of the Guidelines.

Discussions with healthcare providers, which included board-certified physicians, physician assistants and nurse practitioners in family practice and pediatrics, an allergist, and immunologist, shed light on treatment challenges, use of CPGs, and tools they would find helpful in practice. Treatment challenges include insurance coverage; treatment adherence, particularly once symptoms are controlled; patients or caregivers with a poor understanding of asthma and its medications; poor device technique; and allergen management at home. When evaluating whether to apply a guideline to a patient who was outside of the guideline’s age range or severity, providers consider the patient’s history, insurance coverage, and likely adherence, treatment costs, and their prior experience using the treatment and its outcomes. Few providers report having guidelines integrated into their EHR systems and most refer to CPGs as needed by searching the Internet. They also stated that algorithms and decision-support tools were useful.

When asked to review existing EPR-3 materials, most providers reacted positively to the stepwise approach, though some thought it is too complex and suggested adding content that provides guidance on topics such as:

- How to verify a diagnosis of patients presenting as diagnosed
- When to refer to a specialist
- When to consider skin testing
- How to sensitively raise medication side effects, such as oral thrush
- How to monitor and adjust height/weight information in the parameters section of the pulmonary function test as children grow
- Strategies to address patient adherence
- How to talk to patients about monitoring their asthma

### A 360° approach to guideline dissemination for improved implementation

Bringing awareness to the guideline updates through a robust communications strategy has been a priority for NHLBI and the *Learn More Breathe Better®* program. A stakeholder webinar announced the release of the Focused Updates, and a new website was launched providing an overview of the updates, FAQs, and a digital toolkit for healthcare professionals. The digital toolkit includes a Clinician’s Guide and an At-A-Glance Guide, fact sheets on each of the six focused update areas, traditional and social media resources, and links to professional education and continuing medical education (CME) opportunities. NHLBI and *Learn More Breathe Better®* have promoted the Focused Updates through a variety of channels including announcements and information in scientific publications; media activities such as briefings; a press release from the National Institutes of Health; and outreach to top-tier consumer and medical trade outlets. NHLBI has partnered with professional associations to provide CME presentations, webinars, and podcasts and created a suite of digital and social media assets to promote the updates and what they mean for asthma care to patients and providers. Between December 2020 and July 2022, the 2020 Focused Updates report was downloaded 53,048 times and ancillary materials were downloaded 55,319 times. A limitation of these metrics is that repeat downloads cannot be tracked. While this content is considered popular and freely available for public download and use, these metrics are not meant to correlate with successful implementation of the recommendations.

## Conclusion

We developed evidence-based CPG updates for asthma management focused on six topic areas. The guideline development processes and activities undertaken sought to enhance implementability by largely focusing on intrinsic factors as described by Kastner, Gagliardi, and others to produce usable, adoptable, and adaptable CPGs. Consideration of extrinsic factors, such as environmental and behavioral factors, was beyond the scope for this work. The 2020 Focused Updates project included some initial steps toward the proposed future state of redesigning guideline development and implementation by including a multidisciplinary panel, inviting public comments on the draft updates, seeking stakeholder input, creating patient and professional education resources, and developing computable derivative products to enhance guideline uptake and usability in clinical settings. As developers begin to modularly update living guidelines and incorporate more aspects of the proposed future state of living guidelines, it should become easier to streamline processes and shorten the time required to develop new or update existing guidelines. This should enhance the likelihood that CPGs will be implemented and used in practice, ultimately improving the quality of medical decisions and patient care and positively impacting public health.

## Data Availability

Not applicable. The asthma guidelines discussed in the manuscript are available in the public domain.

## Abbreviations

AHRQ: Agency for Healthcare Research and Quality
CDC: Centers for Disease Control and Prevention
CDS: Clinical decision support
CME: Continuing medical education
CPG: Clinical practice guideline
CQM: Clinical quality measure
EHR: Electronic health record
EPR-3: Expert Panel Report 3
FHIR: Fast Healthcare Interoperability Resources
GRADE: The Grading of Recommendations Assessment, Development, and Evaluation
NHLBI: National Heart, Lung, and Blood Institute

## Footnotes

1 The six topic areas addressed by the Focused Updates are intermittent inhaled corticosteroids, long-acting muscarinic antagonists, indoor allergen mitigation, immunotherapy in the treatment of allergic asthma, fractional exhaled nitric oxide testing, and bronchial thermoplasty.
